# Medium-Voltage AC Cable Joints: A Review of Testing Methods, Standards, and Emerging Trends

**DOI:** 10.3390/s25133843

**Published:** 2025-06-20

**Authors:** Alessandro Mingotti, Farshid Babaei, Roberto Tinarelli, Lorenzo Peretto

**Affiliations:** Department of Electrical, Electronic, and Information Engineering (DEI), University of Bologna, 40136 Bologna, Italy; farshid.babaei@studio.unibo.it (F.B.); roberto.tinarelli3@unibo.it (R.T.); lorenzoperetto@unibo.it (L.P.)

**Keywords:** sensors, cable joint, medium voltage, instrumentation, testing, accuracy

## Abstract

**Highlights:**

**What are the main findings?**

- Complete review of standards on MV AC cable joint testing.
- Complete review of the literature and emerging trends on MV AC cable joint testing.

**What is the implication of the main finding?**

- Discussion about the current situation on the testing of MV cable joints.
- Discussion about the key tests to be added/improved in the standard.
- Discussion about the main factors affecting the performance of MV cable joints.

**Abstract:**

Cable joints (CJs) are essential components of power systems, enabling cable network extension and repair. Their design and installation are critical to ensuring reliability. This paper reviews the international standards, state-of-the-art literature, and emerging trends in medium-voltage (MV) AC cable joint testing. It provides a comprehensive overview of existing testing methods, highlighting innovative approaches. The review covers key international standards for CJ testing, both during design and final manufacturing stages. Additionally, it examines the literature on tests developed for assessing factors affecting CJ performance, including temperature, partial discharges, and tangent delta measurements. Recent advancements in artificial intelligence for CJ testing are also discussed. This work aims to present a thorough perspective on current practices and future directions in MV cable joint testing and diagnostics.

## 1. Introduction

Medium-voltage (MV) cable joints (CJs) are critical components in power distribution networks, ensuring the seamless transmission of electrical energy to consumers. Their performance directly impacts service continuity, power quality, and network resilience [1]. Failures in CJs can lead to unplanned outages, costly maintenance, and disruptions affecting residential, commercial, and industrial users. Given that electricity is fundamental to economic activities and daily life, ensuring a stable and uninterrupted supply is a top priority for utilities and grid operators [2].

CJs primarily connect cable segments, enabling network expansion and facilitating repairs [3]. Additionally, they ensure mechanical stability, electrical insulation, and shielding continuity, all of which are essential for safe and efficient operation.

Despite their essential role, ensuring CJ reliability poses significant challenges. Their complex structure consists of multiple layers, including conductive screens, insulation materials, and semi-conductive components, all of which must be properly aligned and installed to prevent defects. Compatibility issues arise when connecting cables from different manufacturers or those with varying insulation technologies and aging conditions. Moreover, the presence of material interfaces introduces potential weak points where partial discharges (PDs), moisture ingress, and mechanical stress concentrations can lead to failures [4].

MV cable networks are particularly vulnerable to environmental and operational stresses. These include thermal cycling due to load variations, mechanical vibrations, and external contaminants, all of which contribute to insulation degradation and electrical treeing. A notable challenge is the increased failure rate of underground CJs in urban areas during summer periods. High ambient temperatures and elevated cable loads can cause soil drying, reducing thermal conductivity and leading to joint overheating and premature aging [5,6].

The expansion of underground cable networks continues to grow, particularly in urban areas where overhead lines are not feasible. While underground cables provide greater reliability and reduce visual pollution, CJs remain weak points in the network due to their exposure to multiple stress factors. Their underground location further complicates maintenance efforts (the CJ cost becomes negligible compared to the replacement procedure one). Addressing these challenges requires advanced condition monitoring techniques [7] to enable early fault detection, proactive maintenance, and optimized intervention strategies [8]. Traditional diagnostic methods such as withstand voltage tests and insulation resistance measurements provide basic assessments but may not detect early-stage defects. More advanced approaches, including PD detection, dielectric spectroscopy, and tangent delta (tanδ) measurements, have become standard practices for evaluating insulation health. Emerging technologies, such as artificial intelligence (AI) and data-driven monitoring [9], are paving the way for predictive maintenance and real-time fault detection, helping utilities anticipate failures before they cause service interruptions [10]. Overall, this paper presents a comprehensive review of AC MV cable joint testing, covering both established standards and recent advancements. By analyzing conventional and emerging techniques, the study aims to provide insights into best practices, key challenges, and future directions in CJ diagnostics and performance evaluation. From the review, the most impacting influence quantities affecting CJs emerge. Furthermore, to the authors’ knowledge, no other document in the literature provides such a comprehensive review on the topic. Hence, the document might be of help to novices of CJs, experts, and researchers. The paper is structured as follows. Section 2 introduces the CJ, its structure, and its characteristics. The major relevant standards, dealing with CJ testing, are analyzed in Section 3. The state of the art and the emerging ideas are addressed in Section 4. In Section 5, the previous sections are discussed and the key information extracted. Conclusions are given in Section 6.

## 2. The Cable Joint

### 2.1. Introduction

**Figure 1:**
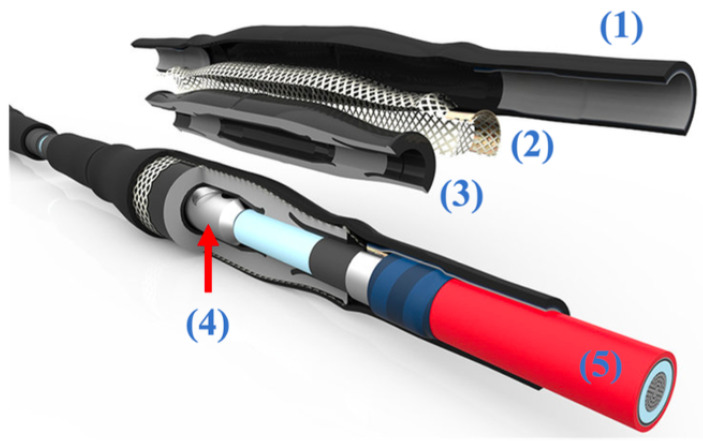
Example of cable joint and its parts. Courtesy of REPL [11].

A cable joint is a mechanical device used to physically connect two sections of cable. To achieve this, the cables must be prepped appropriately before being joined. The structure of a CJ is depicted in Figure 1, with each numbered part corresponding to a specific component. Starting with part (5), this represents one of the two cables that have been prepared for connection. Part (4) is a metallic connector that links the conductors of the cables, carrying the current flowing through them. Part (3) refers to the first layer of insulation and semiconductive materials, which encase the conductive components and ensure proper insulation. Part (2) is the metallic mesh, which restores the ground connection between the two cable sections while also providing mechanical protection to the joint. Finally, part (1) is the cold shrink layer, which is the outermost insulation. This layer is installed without the use of heat or external flames, as the cold shrink process does not require any additional thermal energy.

### 2.2. Cable Joint Categories

CJs are generally classified into several categories based on their construction and insulation type. These categories are provided for convenience and do not encompass all possible joint designs. Some joints may incorporate features from multiple categories, which include the following

- Extruded Joints: These joints connect cables insulated with extruded dielectric materials, with voltage ratings ranging from 2.5 kV to 500 kV.
- Laminated Joints: These joints are used with cables that have a dielectric composed of fluid-impregnated paper, paper/synthetic laminated tape, or varnished cloth.
- Transition Joints: These joints connect an extruded dielectric cable to a laminated dielectric cable, allowing for compatibility between different insulation types.

### 2.3. Cable Joint Construction

CJs can be further classified based on their construction method and installation process, which are outlined as follows:

- Field Vulcanized Joints: Constructed on-site using externally applied heat and pressure to cross-link the joint’s polymeric materials.
- Filled Joints: Feature an outer shell filled with an insulating material to occupy the space around the insulated conductor(s).
- Heat-Shrink Joints: Supplied as expanded polymeric components that shrink when heated, forming a secure seal around the cable. Electrical testing cannot be performed on this joint type before installation, as the stress control, insulation, and shield layers are not integrated during molding or extrusion.
- Multi-Component Cold-Shrink Joints: Consist of two or more pre-expanded components that contract over the cable when their supporting cores are removed. Like heat-shrink joints, these cannot undergo production electrical testing before installation, as the key layers are not integrated during manufacturing. See also single-component cold-shrink joints.
- Pre-Molded Joints: Factory-molded into their final shape before installation, these joints are installed by sliding them over the prepared cable. Unlike heat-shrink and multi-component cold-shrink joints, pre-molded joints allow for production electrical testing because their stress control, insulation, and shield layers are integrated during molding.
- Single-Component Cold-Shrink Joints: Supplied as a single pre-expanded component that contracts onto the cable when its supporting core is removed. Unlike multi-component cold-shrink joints, these can undergo production electrical testing, as their key layers are integrated during molding or extrusion.
- Taped Joints: Constructed on-site using one or more layers of tape applied over the cable insulation. Heat may or may not be used during installation. Like heat-shrink and multi-component cold-shrink joints, taped joints cannot undergo production electrical testing before installation.

## 3. Review of Standards

Compliance with established standards ensures the integrity, safety, and performance of CJs before deployment in power systems. These standards define structured tests to assess insulation integrity, mechanical strength, thermal endurance, and resistance to PDs under simulated conditions. Rigorous testing identifies design, material, or installation weaknesses, ensuring reliability and resilience to operational stresses. Standardized evaluations minimize failures, enhance safety, and support efficient power delivery. Compliance also fosters confidence among manufacturers, utilities, and end users by guaranteeing comprehensive assessment, reducing energy losses and lowering maintenance needs. The following sections briefly outline the most relevant standards for cable joints and summarize their associated tests.

### 3.1. IEEE Std 404^TM^-2022

The standard [12] defines electrical ratings and testing requirements for extruded and laminated dielectric shielded CJs rated from 2.5 kV to 500 kV. It specifies protocols to assess joint performance at the prototype level or final product level, including voltage withstand, dielectric integrity, and cyclic aging. Table 1 outlines the key tests for cable joints as detailed in [12], evaluating their performance, durability, and safety. These tests form the foundation for the analysis of industry standards and the development of improved methodologies.

### 3.2. BS EN 61238-1

This standard [13] applies to compression and mechanical connectors for power cables with rated voltages up to 36 kV (max 42 kV), which are used in applications like buried cables or those installed in buildings. These connectors are for cables with copper conductors (≥10 mm^2^) or aluminium conductors (≥16 mm^2^) and a maximum continuous conductor temperature of 90 °C. The standard does not apply to connectors for overhead conductors or separable connectors with sliding contacts or multi-core connectors. Table 2 summarizes the main tests and the respective purpose for each test.

### 3.3. IEC 60502-4

The standard [14] specifies the test requirements for type testing accessories used with power cables rated from 3.6/6 (7.2) kV to 18/30 (36) kV, as are in compliance with the other IEC 60502 documents. It excludes accessories for special applications, such as aerial, submarine, ship, or cables used in hazardous environments (explosive, fire-resistant, or seismic conditions). Once tests are successfully completed, they do not need to be repeated unless there are changes in the materials, design, or the manufacturing process that could affect performance. Table 3 summarizes the main tests and the respective purpose for each test. $U_{0}$ is the rated power frequency voltage between conductor and earth or metallic screen for which the cable is designed.

### 3.4. IEC 61442

This document [15] defines the test methods for type testing accessories used with power cables rated from 6 kV (max 7.2 kV) up to 36 kV (max 42 kV). The specified test methods apply to accessories designed for extruded and paper-insulated cables in accordance with IEC 60502-2 and IEC 60055-1, respectively. Table 4 summarizes the main tests and the respective purpose for each test.

### 3.5. IEEE Std. 48

This standard [16] provides comprehensive procedures for evaluating cable terminations used in medium- and high-voltage (HV) systems. It is worth mentioning here, because it has been harmonized with [12] in terms of tests to be performed on the terminations.

### 3.6. IEEE Std. 592

This standard [17] outlines performance requirements and test procedures for insulation shields on MV (15 kV–35 kV) CJs and separable connectors. Its purpose is to ensure that these accessories meet safety, durability, and operational reliability under normal and fault conditions. It covers three critical tests—shield resistance (see Table 5), simulated touch current, and fault-current initiation—that are essential for verifying the safety and performance of insulation shields in MV cable joints.

### 3.7. BS HD 629.1

This document [18] specifies performance requirements for type tests for cable accessories for use on extruded insulation power cables. It covers a range of accessories, including indoor and outdoor terminations of all designs, such as terminal boxes. It also applies to various types of joints, including straight joints, branch joints, stop ends, and loop joints, which are designed for use underground, indoors, or outdoors. However, it is important to note that tests specifically addressing ultraviolet and outdoor weather resistance are not included.

### 3.8. BS HD 629.2

This document [19] specifies performance requirements for type tests for cable accessories for use on impregnated paper insulation power cables. Except for some minor modifications, the suggested tests are those indicated in Table 6.

### 3.9. IEC 60230

This document [20] defines the procedure for conducting withstand lightning and switching impulse tests, as well as superimposed impulse tests, on cables and their accessories. It focuses solely on the test methods independent of the selection of test levels. It outlines the characteristics and conditions of the test installation, procedures for performing withstand impulse tests, and methods for conducting tests above the withstand level for research purposes. The document mainly refers to the IEC 60060 family of standards for the voltage to be used. The core of the document can be found in the annexes in which tests conditions, calibration methods, and measurement setups are briefly explained.

### 3.10. IEC 60055-1

This standard [21] defines test procedures for impregnated paper-insulated metal-sheathed cables, excluding gas-pressure and oil-filled cables, with rated voltages from 0.6/1 kV to 18/30 kV. Despite applying to a type of cable that is being replaced day after day, the standard is still valid. It also includes type tests for accessories used with cables rated from 3.6/6 kV to 18/30 kV. Testing paper-impregnated insulated cables is more challenging. This is confirmed by the recent literature attention to other types of cables. Therefore, the focus is not kept on paper-impregnated insulated cables.

### 3.11. National Regulation

The reader should note that national documents with specifications are typically developed by manufacturers or operators and are only valid within their country of origin. In this research, such documents are not discussed, as they cannot be generalized and, most importantly, are often not available in English.

## 4. Literature Review

**Figure 2:**
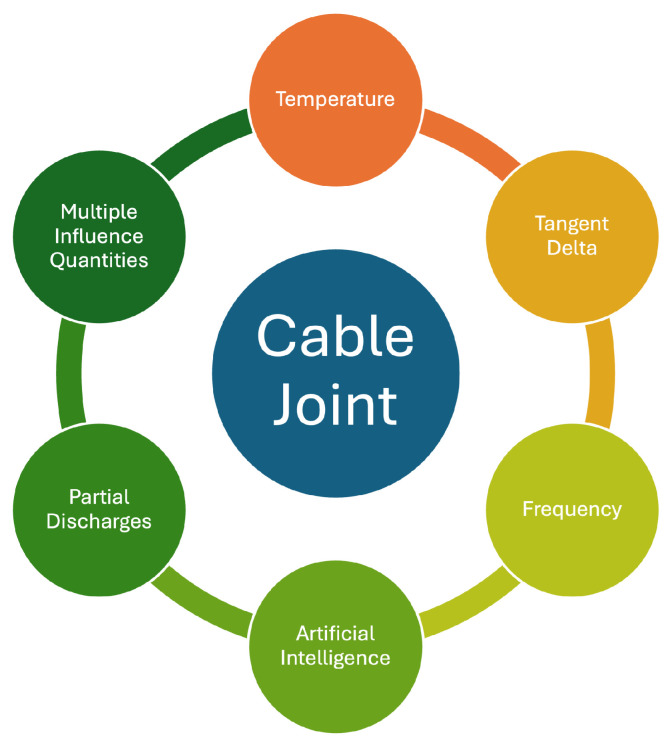
Topics addressed in the literature review.

This section presents a comprehensive review of the current literature on MV cable joint testing. It is structured by topic to enhance reader comprehension. The topics are summarized in Figure 2.

### 4.1. Temperature

Temperature is one of the most influential factors affecting CJs. Daily and seasonal temperature variations impact the insulating materials within the system. Therefore, studies and tests on temperature effects are highly significant.

#### 4.1.1. Thermal Behavior and Temperature Distribution

Bragatto et al. [5] investigated the thermal effects of ground faults, showing that poor contact resistance between cable screens and copper stockings during cross-country faults can lead to overheating and cable failure. Zhao [22] used ANSYS simulations to study the temperature distribution on the outer surface of CJs, revealing that defects like gaps and water droplets cause uneven temperature distribution. Di Sante et al. [23] examined how thermal cycles affect the interfacial pressure between XLPE and silicone rubber in CJs, contributing to aging and potential failure due to void formation. Bragatto et al. (2023) [24] assessed the thermal behavior of cold-shrinkable MV joints through simulations and real-world measurements, concluding that cable current profiles influence joint temperatures more than ambient temperature. In Bragatto et al. [25] is presented a 3D thermal model of underground MV cables and joints, validating it with field measurements to understand overheating and failure risks under different environmental conditions. In their 2017 work, Bragatto et al. [26] developed a nonlinear thermal circuit model for MV cable joints, which accurately predicts internal temperatures during both steady-state and transient conditions.

#### 4.1.2. Aging, Diagnosis, and Monitoring

Papers in this category investigate the failure modes, causes of damage, and diagnostic methods for cable joints. The works of Peretto et al. [27] and Sturchio et al. [28] discuss the deployment of sensors to monitor current, pressure, temperature, and humidity in MV cable joints, helping assess the aging rate of their insulation. The work of Mingotti et al. [29] describes a low-cost, modular monitoring unit for MV cable joint diagnostics, providing an affordable approach to real-time monitoring in distribution networks. Kong et al. [30] analyzed CJ faults using simulation control methods, focusing on temperature field distribution and the effectiveness of protection strategies. Ruan et al. [31] proposed a model using support vector regression to estimate the temperature inside three-core MV cable joints, which was validated by temperature-rise tests, offering a reliable approach for real-time temperature monitoring. Calcara et al. [32] reported on temperature measurement campaigns for MV underground CJs, proposing discharge mechanisms and strategies for improving joint resilience.

Additionally, Di Sante et al. [33] characterized interfacial pressure variations in MV CJs under thermal cycling conditions, providing important insights into how temperature changes impact joint integrity. Mingotti et al. [34] analyzed the variation in MV cable joints’ equivalent impedance with temperature, linking this to their performance and failure modes. Bragatto et al. [35] conducted a year-long measurement campaign to assess the temperature variations within cold-shrinkable MV cable joints, showing that even under extreme conditions, these joints remain free from overheating. Together, these studies underline the importance of environmental monitoring in detecting, diagnosing, and preventing failures in MV cable joints, helping enhance their reliability and longevity in real-world conditions.

#### 4.1.3. Design and Innovation in Cable Joints

Innovative designs in CJs aim to enhance performance, safety, and fault detection capabilities. Wang et al. [36] proposed a new intelligent explosion-proof CJ that integrates fault early warning and location identification, ensuring the cable’s safe operation and providing high reliability. The design of this new joint was validated both through simulations and real tests, demonstrating its superior insulation, waterproofing, and explosion-proof properties compared to traditional designs. Moon et al. [37] investigated the overheating issues in MV aluminum CJs, focusing on joint processing and mechanical splicing as key contributors to thermal resistance. The study proposed an optimized jointing method that balances the removal of substances inside the joint with improved thermal properties, offering a solution to mitigate overheating and enhance network reliability.

### 4.2. Partial Discharges

#### 4.2.1. Introduction

Partial discharges are localized electrical discharges [38] that occur within a portion of the dielectric material between two conductors. They typically arise in small voids within solid insulation, gas bubbles in liquid dielectrics, or at interfaces between materials with differing dielectric properties. PDs can also occur at sharp points or edges of metallic surfaces.

Although these discharges involve relatively small amounts of energy and may not immediately compromise the dielectric strength during standard HV tests, their cumulative effect can gradually degrade the insulation. This deterioration, over time, can lead to complete dielectric failure even under nominal operating voltage. The extent of this degradation depends on the dielectric material, manufacturing processes, electrical stress, and operational conditions.

While PDs were not a major concern in the early 20th century, their relevance increased significantly with the introduction of new insulating materials, the drive for compact designs, and the use of higher operating voltages. Today, PD measurements are standard practice in both type and acceptance testing, as well as in-service diagnostics for MV and HV equipment [39], to identify weaknesses before irreversible damage occurs.

PDs can be classified into three main types [40]:

- Internal discharges, including treeing, are the most common cause of insulation failure. These originate in gas-filled voids within the dielectric, where the electric field becomes intensified. Once a discharge occurs, residual surface charges can distort the field and trigger future discharges at varying locations and voltages. Treeing, particularly relevant in extruded polyethylene cable insulation, involves microscopic discharge channels branching through the dielectric due to localized charge injection and destabilization, leading to rapid breakdown once initiated.
- Surface discharges happen along interfaces between different dielectrics where a strong electric field is present parallel to the surface. These discharges can spread over the material surface, causing tracking and erosion that may culminate in complete breakdown.
- Corona discharges arise near sharp metallic protrusions in highly divergent electric fields. In air, a corona discharge produces ozone and nitrogen compounds that, in the presence of moisture, corrode metallic surfaces. This leads to conductive paths and eventual insulation failure. Corona discharge is especially problematic in $SF_{6}$-insulated systems, where the byproducts can attack dielectric surfaces. Moreover, corona discharge is a significant source of noise during PD testing, so it is essential to avoid sharp edges in the test environment by rounding all protrusions.

**Figure 3:**
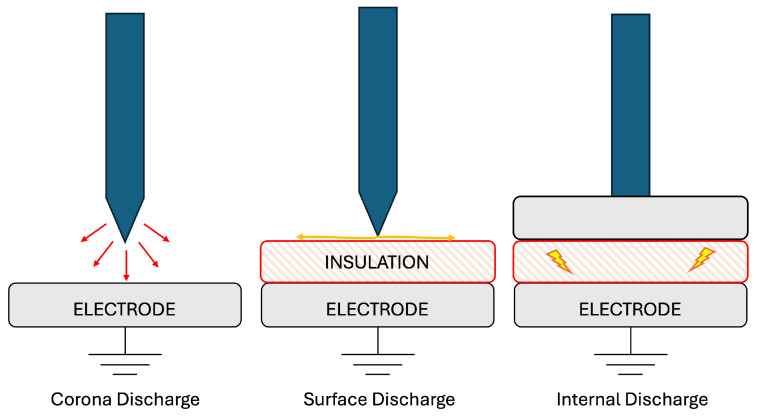
Types of PDs.

Figure 3 helps visualize the types of discharge.

While internal, surface, and corona discharges were introduced in general terms, it is indeed important to clarify how these phenomena specifically arise within CJs. Internal discharges typically originate within voids or air gaps that may be inadvertently introduced into the insulation material during manufacturing or installation, particularly due to improper compaction or imperfections in the jointing process. Surface discharges occur along interfaces between different dielectric materials, often exacerbated by the presence of contaminants or moisture ingress, especially at the boundaries between insulation and stress control elements. Corona discharges, on the other hand, are associated with localized electric field enhancements and may arise around the metallic parts within the CJs.

#### 4.2.2. PD Propagation

The propagation PD signals within branched cable networks presents significant challenges due to the complex nature of signal transmission and the variations in impedance at junctions. According to M. Shafiq et al. [41], understanding the way PD signals split at T/Y splices is essential for improving condition monitoring in medium-voltage cable feeders. The study highlights how characteristic impedance influences energy division at the joint, affecting diagnostic accuracy. Building on this, F. Zhao et al. [42] examined how scattering parameters play a crucial role in signal attenuation and distortion as PD pulses travel through the joint. Using both computational simulations and experimental measurements, the study reveals that higher frequencies lead to increased attenuation and signal fluctuation, further complicating PD detection. By analyzing the interplay between phase velocity, attenuation constant, and frequency, [42] provides valuable insights into the fundamental challenges of PD signal interpretation in cable networks.

#### 4.2.3. PD Detection Techniques and Sensitivity Analysis

Generally speaking, a PD can be obtained with two primary techniques: online and offline testing. Each technique has distinct advantages and limitations. Online methods involve monitoring PD activity while the cable/CJ remains energized and operational. They allow for real-time detection of insulation degradation without service interruption, making them ideal for critical systems requiring continuous operation. Online testing facilitates early fault detection and can be integrated into condition monitoring systems. However, it is susceptible to electrical noise, which can obscure low-level PD signals, and requires specialized equipment and expertise to mitigate interference. Offline PD testing, in contrast, is conducted with the cable de-energized and disconnected from the system. This approach allows for controlled testing conditions, enabling the application of various voltage levels to assess insulation performance comprehensively. Offline testing provides higher sensitivity and accuracy, as it is less affected by external noise, and can detect defects that may not manifest under normal operating conditions. However, it requires system downtime and may not capture transient PD events occurring during regular operation. In practice, a combination of both online and offline PD testing methods is often employed to achieve a comprehensive evaluation of cable insulation health, balancing the need for continuous monitoring with detailed diagnostic analysis.

Detecting PDs in CJs requires highly sensitive diagnostic tools that can accurately capture and analyze discharge events. J. Ruan et al. [43] explored the use of capacitive couplers in PD detection, demonstrating how their sensitivity depends on factors such as coupling electrode width and defect type. Their findings indicate that capacitive couplers are particularly effective in tracking PD trends over time rather than quantifying absolute discharge levels. In contrast, R. Wu and C. Chang [44] presented a different approach to PD monitoring, employing a peak detect circuit and a digital signal processor to convert PD signals into discharge sequence data. This method allows for real-time assessment of cable joint conditions, enhancing predictive maintenance strategies. Meanwhile, X. Zhang et al. [45] provided a comprehensive review of PD detection techniques, comparing offline and online methods. Barbieri et al. [46] proposed an improved sensor for early detection and localization of PDs in CJs, which are known weak points in power systems. Tested on artificially defected joints, the sensor successfully detected PD activity and showed alignment with simulations and historical data, offering a promising tool for online condition monitoring. Offline testing, though highly accurate, requires system shutdowns, whereas online monitoring enables continuous assessment but faces challenges such as noise interference and signal attenuation. Understanding the strengths and limitations of each technique is crucial for selecting the most effective diagnostic approach.

#### 4.2.4. PD Characteristics Under Different Conditions

The nature of PD behavior varies significantly depending on the type of defect present within the cable joint. Y. Sun et al. [47] investigated stress cone dislocation defects, revealing how variations in dislocation length alter the electric field distribution and impact PD initiation. Their findings suggest that applying semi-conductive self-adhesive tape around the exposed XLPE insulation layer can significantly improve joint reliability and extend operational life. H. Yang et al. [48] expanded on this by analyzing the effects of various defect types, such as needle damage and insulation scratches, on the electrical field within cable joints. Their finite element analysis (FEA) showed that needle damage induces the most severe field distortions, increasing the likelihood of PD occurrence. Similarly, N. G. Li et al. [49] examined the impact of defect severity under oscillating voltage conditions, demonstrating that as defect severity increases, PD frequency and discharge intervals expand. Furthermore, B. Rajalakshmi et al. [50] explored the influence of different insulation materials on PD inception voltage. Their study concluded that epoxy resin insulation offers superior PD resistance compared to other materials, reinforcing its suitability for high-reliability applications.

#### 4.2.5. Simulation and Experimental Studies on PD Behavior

Numerical simulations and laboratory experiments provide critical insights into the mechanisms governing PD activity in CJs. Y. Xia et al. [51] conducted experimental tests on 10 kV XLPE-insulated cables, identifying six typical defects—including dents, knife wounds, and semi-conductive fractures—that contribute to significant local discharge phenomena. Their results emphasize the importance of defect characterization in PD diagnostics. N. G. Li et al. [49] complemented these studies by applying statistical analysis to PD localization, demonstrating how discharge intervals and magnitudes correlate with defect severity. Lee et al. [52] presented field testing results on PD measurements of 22 kV MV cables using a DAC and sinusoidal very low frequency (VLF). In 78 circuits tested, the DAC identified up to 25% more PD events, with only 30–45% overlap between methods. The DAC showed better alignment with 50 Hz AC in PD inception voltage, supporting previous studies and highlighting the limitations in VLF comparability. Their findings underscore the value of advanced statistical techniques in refining PD assessment methodologies.

#### 4.2.6. PDs and AI

As the field of PD detection continues to evolve, researchers are increasingly turning to computational intelligence techniques to enhance diagnostic accuracy. S. Govindarajan et al. [53] discussed the integration of deep learning and other AI-based approaches in PD localization, highlighting their potential to automate fault detection and improve predictive maintenance. Similarly, X. Zhang et al. [45] identified emerging trends in PD measurement, such as the use of high-frequency sensors and machine learning (ML) algorithms to refine signal interpretation. These advancements address longstanding challenges, including signal attenuation and interference. Finally, M. Shafiq et al. [41] emphasized the importance of developing integrated condition monitoring systems that encompass not only CJs but also transformers, terminations, and entire power distribution networks. By leveraging advanced PD diagnostics, utility companies can enhance grid reliability and optimize maintenance strategies for MV infrastructure.

### 4.3. Tangent Delta

#### 4.3.1. Introduction

Tanδ measurements are used to assess the amount of real power loss occurring within a dielectric material [54]. By comparing the measured tanδ value to a known reference for the specific type of dielectric being tested, the condition of the system can be evaluated based on how much the dielectric loss deviates from the expected norm. In cable systems, these reference values can be drawn from several sources, such as measurements from adjacent phases, cables of the same design and age within the same installation, or even from values recorded when the cable was new. Long-term trends and variations during a single measurement, as well as changes in response to applied voltage or frequency, can also provide valuable reference points. Additionally, industry standards and experience-based data libraries serve as important benchmarks.

Tanδ, also known as the Dissipation Factor (DF), is determined by applying an AC voltage and analyzing the phase difference between the voltage waveform and the resulting current waveform. This phase angle is then used to break the total current into two components: the charging current ($I_{C}$) and the loss current ($I_{R}$). Tanδ is defined as the ratio of these two, specifically the loss current divided by the charging current.

**Figure 4:**
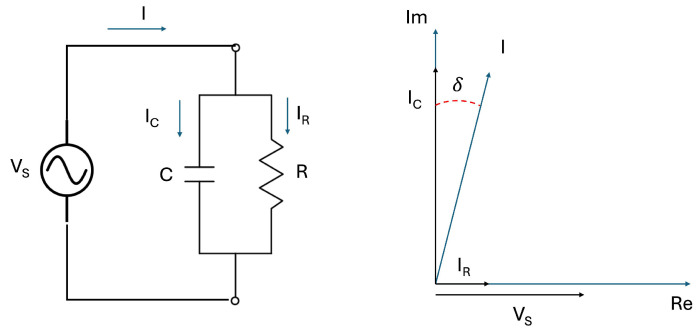
Tanδ measurement circuit (**left**) and its vector diagram (**right**).

An equivalent circuit model of a cable can help visualize this concept (see Figure 4), which is typically represented as a capacitor (C) in parallel with a resistor (R). At a given frequency (ω) and voltage ($V_{S}$), the tanδ value corresponds to the ratio between the resistive and capacitive currents as

(1) $$\tan\delta =\frac{I_{R}}{I_{C}}=\frac{\frac{V_{S}}{R}}{\frac{V_{S}}{\frac{1}{\omega C}}}=\frac{1}{\omega RC}.$$

The effectiveness of tanδ testing is greatly enhanced when the specific cable and its associated components are well identified. This allows for a direct comparison between the measured values and those expected for known materials or previously recorded values for the same system. Note, when it comes to evaluating individual components like CJs, the technique faces significant limitations. This is because tanδ provides a global measure of dielectric losses across the entire cable system under testing rather than localizing the measurement to specific components. CJs constitute a very small portion of the total cable length and insulation volume, so their individual contribution to the overall tanδ value is minimal, often falling within the margin of measurement uncertainty. Moreover, the capacitive and resistive characteristics of the joint are effectively “masked” by the dominant properties of the much longer cable sections. As a result, even if a joint is degraded or defective, its influence may not be detectable unless the damage is severe enough to affect the global dielectric losses. This makes tan delta measurement a challenging tool for isolating joint-specific defects and highlights the need for complementary techniques like partial discharge testing for localized diagnostics. In what follows, the description of techniques and methods typically refers to cables. However, specific techniques dedicated to CJs are reported and explicitly indicated.

#### 4.3.2. Measurement Techniques

The basic measurement techniques, which create the state of the art, are given in [54]. Other techniques, generally applicable to cables, are, for example, the following. Morsalin et al. [55] analyzed the dielectric behavior of service-aged XLPE cables using the polarization and depolarization current (PDC) method. By applying Daniel’s normalization and transforming PDC data into the frequency domain, they provided a detailed interpretation of loss mechanisms and their contribution to the overall dissipation factor under varying test conditions. Chi et al. [56] proposed a condition assessment method for XLPE cables based on signal propagation characteristics, enabling the estimation of dielectric loss using only voltage measurements. Their approach, supported by cable modeling and simulations, demonstrated effective performance across different cable lengths and insulation states. Arikan et al. [57] focused on predicting dielectric parameters during accelerated aging of MV XLPE cables, comparing curve fitting, decision tree, and artificial neural networks (ANNs) techniques. Among these, the ANNs achieved the highest accuracy, offering a promising tool for forecasting insulation performance and reducing diagnostic testing time. Finally, being specifically developed for MV CJs, Mingotti et al. [58] designed a test setup for tanδ measurements on MV cable joints, utilizing calibrated resistors under 1 kV and 50 Hz conditions.

#### 4.3.3. Tangent Delta vs. Influence Quantities

Ghaderi et al. [59] expanded the previous work by evaluating temperature effects on tanδ, confirming its role in fault prediction. Ghaderi et al. [60] demonstrated the correlation between external mechanical pressure and tanδ values, where increased pressure led to a decrease in tanδ, extending joint lifespan. Permal et al. [61] proposed a high-frequency alternating current technique to isolate tanδ responses of CJs from bulk cable measurements, improving maintenance precision. This differentiation is crucial for effective CJ monitoring.

### 4.4. Frequency-Based Measurement Techniques

This section aims to focus on the testing frequencies and the relevant techniques. Over the entire spectrum, different behaviors can be extracted and meaningful conclusion extracted. Low- high-frequency testing and signal processing techniques are described.

Sukdev et al. [62] studied the influence of test voltage frequency on electric field stress distribution in MV power CJs. Their FEA simulations compared behaviors under 50 Hz and 0.1 Hz VLF conditions, revealing significant changes in the electric field profiles. The results suggest that VLF tests may not be as reliable as 50 Hz tests for evaluating power cable accessories. Yang et al. [63] developed a finite-difference time-domain model to study radio frequency (RF) PD signals in CJs. The transfer functions exhibited peaks at 380 MHz and 790 MHz, with variations depending on source location and direction. Additionally, Hou et al. [64] proposed an enhanced time domain reflectometry (TDR) and multi-ratio measuring system to locate long-distance distribution CJs with high accuracy, improving the detection of potential defects. Maier et al. [65] proposed a high-frequency simulation model to compute transmission line parameters of MV cable joints, enhancing existing models to account for field regulation in HV joints. Similarly, Norouzi et al. [66] investigated the effectiveness of frequency domain analysis (FDA) in detecting cable joint locations with improved sensitivity compared to TDR techniques. Fritsch et al. [67] analytically determined the measurable bandwidth of PD in MV cables using a simulation model, revealing significant signal attenuation and dispersion during transmission. Their findings show that most PD signals arrive with a bandwidth below 10 MHz, supporting the development of cost-effective, bandwidth-optimized PD detectors.

### 4.5. Multiple Influence Quantities

#### 4.5.1. Introduction

The performance and diagnostic accuracy of condition monitoring techniques in power systems are significantly affected by a wide range of influence quantities. These can be broadly categorized into environmental factors—such as temperature, humidity, and moisture ingress—and operational or system-related factors, including electrical frequency, load cycles, and aging. While the effects of individual influence quantities are often studied in isolation, their simultaneous presence can create complex interactions that alter dielectric behavior, measurement reliability, and the progression of insulation degradation. This issue is especially critical in CJs, which are more vulnerable than continuous cable sections due to their complex construction, heterogeneous materials, and susceptibility to mechanical and environmental stressors. In CJs, the co-occurrence of stressors can, for example, accelerate PD activity and reduce detection thresholds, making early diagnosis both more challenging and more essential. On the contrary, some influence quantities might have a positive effect on the accuracy of the CJ. Hence, the combination of more than one influence quantity does not always have a negative impact (but it remains not predictable). Despite this, the combined effects of multiple influence quantities on CJ performance remain mostly underexplored, pointing to an urgent need for comprehensive studies that consider the interaction between thermal, moisture, and electrical stress factors. In what follows, such aspects are discussed, and the relevant studies are mentioned.

#### 4.5.2. PD and Temperature

The study by J. Borghetto et al. [6] investigates failure causes in MV cable joints, particularly during summer, through a multi-year experiment. Twelve cables with different joint types were connected in a short-circuited ring and energized with rated voltage. The degradation of joints was monitored using PD measurements, Frequency Dielectric Spectroscopy, and DF techniques. The results highlight the effectiveness of these diagnostic tools in identifying failure mechanisms under simulated summer conditions. In their 2019 study, J. Borghetto et al. [68] compared online and offline PD measurements on thermally aged MV cable joints. Twelve cables underwent daily thermal cycles, with PD activity monitored during heating and cooling. The study found that online PD monitoring detected defects earlier, while offline tests identified them after several thermal cycles, showing that online monitoring is more effective for early fault detection in thermally stressed cables. Pompili et al. [69] investigated the vulnerability of MV cable joints to PDs, especially under elevated temperatures, which lower PD inception voltage and increase failure rates—particularly in summer. The paper proposed a testing method that assesses joint quality by measuring PD inception voltage across varying temperatures to identify joints resistant to thermal stress-induced failures.

#### 4.5.3. PD, Frequency, and Humidity

The study from Xu et al. [70] investigated the vulnerability of CJs, particularly in power cables, to moisture intrusion. Using Computer Simulation Technology Studio Suite, the authors developed a simplified 3D model of a 10 kV cold-shrinkable intermediate CJ based on TDR principles. The research analyzed how moisture affects the impedance characteristics and waveform reflections in the joint. As the moisture content increased, the study observed a decrease in impedance and a reduction in the amplitude of reflected waveforms. In more severe cases, opposite-direction pulses appeared. The study introduced two diagnostic approaches—vertical and horizontal comparison—to assess the extent of moisture intrusion and water content in CJs. Gong et al. [71] proposed a short-circuit response method to locate damp cable joints affected by moisture in underground power cables, using impedance discontinuity and frequency domain reflection techniques to improve defect detection efficiency. Anthony et al. [72] developed a set of condition assessment criteria for identifying water ingress in MV underground CJs, combining PD testing, insulation resistance tests, and TDR to detect defects without the need for costly cable replacements. Dreßler et al. [73] conducted long-term evaluations of CJs under water stress, testing their resistance to voltage, water, and mechanical stress and highlighting the impact of poor cable joint sealing on insulation degradation and failure.

### 4.6. Artificial Intelligence

The potential role of AI is to enhance the monitoring and diagnostic capabilities for CJs, which are indeed more prone to insulation defects compared to cable sections due to their complex structure and manual installation processes. AI can be effectively utilized to process large volumes of measurement data, identify patterns indicative of emerging defects, and distinguish between different types of partial discharges (e.g., internal, surface, or corona). By applying ML techniques—such as supervised classification, anomaly detection, or clustering—AI models can learn from historical discharge patterns and predict the likelihood of insulation failure with higher accuracy than traditional threshold-based methods. Moreover, AI facilitates continuous, real-time condition assessment, enabling more proactive maintenance strategies. From the literature, V. Negri et al. [74] designed a scenario to generate data for ML algorithms, testing its application in fault diagnosis for CJs. They found ML effective for predictive maintenance, but it resulted in a few practical challenges to optimize. In another study, H. Jiang et al. [75] used Magnetic Pulse Crimping technology to connect aluminum and copper CJs and analyzed defects such as scratches and inadequate length, revealing significant performance issues such as increased electrical resistance and reduced mechanical strength. Meanwhile, V. Negri et al. [76] compared various ML algorithms for assessing the health of CJs, highlighting the strengths, weaknesses, and performance metrics for selecting the best algorithm. In another study, the challenges of uncertainty in ML training, due to algorithmic uncertainty and knowledge gaps, were analyzed for MV CJ fault prediction by V. Negri et al. [77]. Finally, S. J. Chang and G. Kwon [78] proposed an unsupervised deep learning approach, combining LSTM and VAE, to detect anomalies in CJs, demonstrating better accuracy in early defect detection compared to conventional reflectometry methods. Chang et al. [79] applied convolutional neural networks to classify insulation status in underground CJs using phase-resolved PD patterns, achieving 97.3% accuracy across different defect stages. Louro et al. [80] used ML on real-world indirect observations from smart grids to estimate MV cable network failure types, reaching up to 94% accuracy for cable faults, though joint failures remained challenging to distinguish. These advancements showcase the growing role of AI and deep learning techniques in improving the detection and maintenance of CJs in power systems.

## 5. Discussion

This section analyzes the insights gathered from the reviewed standards and literature, identifying gaps in current testing practices and suggesting directions for future improvements MV CJ diagnostics and standardization.

### 5.1. Discrepancies and Limitations in Existing Standards

The analysis of international standards reveals a structured but heterogeneous landscape. While each standard broadly aims to ensure the electrical, mechanical, and environmental reliability of CJs, several discrepancies in scope, voltage range, testing methods, and performance criteria are evident. Moreover, temperature levels, PD test voltage thresholds, and short-circuit test parameters vary, affecting the comparability of results across standards. Furthermore, we outline the following discrepancies and limitations:

- Current standards are primarily laboratory-based and lack environmental realism. Tests are conducted under controlled conditions that do not account for complex field scenarios such as combined temperature–humidity–cyclic loading or the long-term degradation mechanisms observed in service.
- AI-based diagnostic methods, widely discussed in the recent literature, remain absent from any formal standard. As artificial intelligence becomes increasingly integrated into condition monitoring—enabling pattern recognition, anomaly detection, and predictive maintenance—there is a pressing need to standardize data acquisition, model validation, and interpretability criteria. Without such regulation, the reliability and comparability of AI-driven diagnostics across different utilities and equipment types remain uncertain, potentially hindering broader industry adoption and trust. Future standards should address these issues to ensure the safe, transparent, and effective deployment of AI in MV CJ diagnostics.
- High-frequency behaviors—particularly those influencing PD signal propagation, attenuation, and detection—are critical for the accuracy and effectiveness of modern diagnostic and field monitoring systems. Despite their importance, these aspects are often underrepresented in current standards and testing protocols. A more comprehensive integration of high-frequency phenomena into diagnostic methodologies would enhance the ability to localize defects, distinguish noise from true PD activity, and improve the reliability of both conventional and AI-enhanced monitoring systems. Incorporating these parameters is essential to reflect the real-world electromagnetic behavior of CJs and accessories under operational conditions.

To promote harmonization and ensure consistent qualification procedures, it is recommended to align key test conditions and pass/fail criteria—particularly for partial discharge levels, thermal cycling, and short-circuit endurance—across standards. This would facilitate cross-compatibility in international applications and ensure a uniform level of performance and safety across diverse grid environments. Another suggestion is cross-referencing. It would be helpful to cite a specific document/standard every time that the context requires a reference for the reader in a particular context.

### 5.2. Standard vs. Literature: Insights from Real-World Monitoring

The literature significantly expands upon standard test conditions by examining MV cable joint behavior under realistic and compound stressors. We outline relevant studies and their findings as follows:

- Temperature-related degradation is frequently highlighted in both simulation and experimental work. Studies demonstrate how soil drying, ambient heating, and current loads contribute to thermal runaway—a scenario not realistically captured in most standard cyclic aging protocols.
- PD monitoring in the literature includes sensor innovation, defect classification, propagation analysis at joints, and AI-based detection. Standards, in contrast, remain limited to pass/fail discharge thresholds.
- Tanδ measurements are known to be sensitive to variables such as temperature, pressure, and especially cable length, which significantly influence the overall dielectric loss profile. While research has proposed tailored setups to isolate and assess the contribution of joints specifically, current standards predominantly apply generic criteria at the cable-system level. This approach overlooks the localized nature of joint degradation and limits the diagnostic resolution needed to detect early-stage joint faults. To bridge this gap, future standards should consider joint-specific methodologies and correction factors that account for the disproportionately small yet critical role joints play in the overall insulation system.
- Moisture and humidity ingresses are shown to significantly affect impedance and PD inception. Standards mention humidity or submersion tests but lack the compound multi-stress profiles studied in the literature.

### 5.3. Emerging Diagnostic Trends and Integration Opportunities

The literature introduces forward-looking diagnostic tools, which include the following:

- AI- and ML-based predictive diagnostics, achieving classification accuracies above 90% for fault types, PD severity, and insulation health.
- Modular, low-cost sensor systems enabling real-time, in situ monitoring of MV joints.
- High-frequency analysis tools capable of isolating CJ responses from those of bulk cables, aiding in early defect localization.

To remain relevant, standards should begin incorporating the following:

- Guidelines on the minimum requirements for AI diagnostic validation and data acquisition.
- Testing procedures involving multiple influence quantities simultaneously (e.g., temperature, humidity, electrical cycling, etc.).
- Provisions for high-frequency response testing and signal integrity assessments.

### 5.4. Need for More Realistic Testing Protocols

The literature strongly underscores the inadequacy of current testing protocols in simulating real-world degradation mechanisms. Particularly, the following gaps have been identified:

- Thermal–humidity cycles, typical of underground installations in urban settings, are almost entirely absent in standard type tests.
- Aging under simultaneous mechanical, thermal, and electrical stress is not captured, though it mirrors actual operating environments.
- Field data indicate seasonal failure spikes, especially in summer, due to the compounding effects of load, ambient heat, and soil dehydration. These insights remain unaccounted for in cyclic aging test design.

Hence, future standards should shift toward scenario-based testing, incorporating probabilistic degradation models, and simulation-informed test sequences.

### 5.5. Recommendations for Research and Standardization

This review highlights several opportunities:

1. Initiate inter-standard harmonization to consolidate test scopes and enhance international consistency.
2. Establish AI-readiness annexes in IEEE or IEC documents for condition monitoring of MV cable joints.
3. Define minimum datasets and benchmarking protocols for AI algorithms used in fault prediction.
4. Promote multi-influence testing frameworks, enabling combined temperature, humidity, mechanical, and electrical stress evaluations.
5. Support field-deployable test validations through long-term monitoring campaigns to calibrate lab-based simulations.

### 5.6. Summary

Overall, while international standards provide a solid foundation for cable joint evaluation, they are increasingly outdated relative to the capabilities and insights offered by modern diagnostic technologies and field studies. Incorporating the dynamic, multi-physics phenomena identified in the literature is essential to ensure cable joint reliability in the face of evolving grid demands and environmental stressors.

## 6. Conclusions

This review has provided a comprehensive review of current testing methodologies, international standards, and recent research developments in the field of medium-voltage AC cable joint diagnostics. By systematically comparing regulatory frameworks with the academic literature, the paper highlights both the strengths and limitations of existing testing protocols. While the analyzed standards offer a solid foundation for type testing, they largely reflect idealized laboratory conditions and often fail to capture the complex, multi-physical degradation processes that joints undergo in real-world environments. In contrast, the recent literature introduces innovative diagnostic methods, including partial discharge localization, advanced tangent delta measurements, and the application of artificial intelligence for predictive maintenance. These approaches not only improve defect sensitivity but also support the development of continuous, in-service monitoring strategies. However, their absence in current standards signals a growing gap between academic progress and regulatory adoption. This work is intended to serve as a valuable resource for researchers seeking to identify open challenges and develop novel solutions in medium-voltage cable joint diagnostics. At the same time, it provides system operators and utilities with a critical overview of emerging technologies that could enhance network reliability, reduce unplanned outages, and support cost-effective maintenance planning. By aligning academic advancements with industrial needs, this paper aims to foster the development of more realistic, robust, and forward-looking testing procedures that better reflect the demands of modern power distribution systems. 

## Figures and Tables

**Table 1:** List of tests from IEEE 404-2022.

Test Name	Purpose	Technique	Expected Outcomes
**Dielectric Integrity Test (PD and Ionization)**	Ensures insulation integrity and ionization stability in transition joints.	Partial Discharge Test: Voltage raised 20% above specified level; discharge measured.	Dielectric Integrity Test.
**AC Withstand Test**	Confirm insulation withstand capability under AC voltage.	Voltage applied at a specified rate (5 kV/s ± 3 kV/s) to design or production test levels.	Joint must withstand test voltage without failure for the specified duration.
**Basic Insulation Level (BIL)**	Test impulse voltage withstand capability.	Conducted per IEEE Std 82 using full lightning impulse wave shapes.	Must withstand 10 positive and 10 negative impulses at the specified magnitude.
**Short-Time Current Test**	Assess joint’s ability to endure short-circuit currents.	High current applied to raise conductor temperature to rated short-circuit levels.	No joint damage under current or post-test AC voltage.
**Cyclic Aging Test**	Validate reliability under thermal and load cycling conditions.	30 cycles of alternating current to achieve rated emergency operating temperatures (e.g., 6 h each).	Joints must exhibit no operational degradation or loss of insulation integrity in air or submerged conditions.
**Shielding Test**	Verify shielding effectiveness and resistance to fault currents.	Resistance measurement and fault current initiation per IEEE Std 592.	Shielding must meet specified resistance and fault initiation criteria.
**Jacket Seal Test**	Ensure environmental seals prevent moisture ingress.	Submersion and pressure testing.	No moisture ingress or seal failure under test conditions.
**Connector Thermal Test**	Evaluate thermal stability of connectors under operating conditions.	Thermal cycling and mechanical testing on connectors with varying conductor sizes and materials.	Connectors must retain thermal integrity and mechanical stability.
**High-Voltage Time Test**	Ensure performance over prolonged exposure to high voltage.	Continuous high-voltage application over a specified duration.	No breakdown or failure during test duration.

**Table 2:** List of crucial tests for cable joints from BS EN 61238-1.

Test Name	Purpose	Technique	Expected Outcome
**Heat Cycle Test**	Evaluate thermal stability and resistance consistency of connectors under cyclic heat load.	Current is circulated to elevate connector temperature to equilibrium (120–140 °C). Resistance measured over 1000 cycles.	Stable electrical resistance and temperature performance across cycles; no significant degradation.
**Electrical Resistance Measurement**	Verify stability of electrical resistance under cyclic heat stress.	Resistance measured at 20 °C using direct current.	Stable and repeatable resistance values within defined limits to ensure reliable electrical performance.
**Short-Circuit Test**	Confirm thermal durability of connectors under short-circuit conditions.	Short-circuits applied, raising reference conductor temperature to 250–270 °C.	No permanent deformation, melting, or significant resistance increase; connectors withstand defined fault currents.
**Mechanical Tensile Test**	Ensure mechanical strength of connectors against tensile stress.	Load applied at 10 N/mm^2^/s rate and held for 1 min.	No slippage or failure under tensile force; connectors retain secure attachment to the conductor.
**Maximum Temperature Recording**	Monitor temperature to ensure connectors do not exceed operational temperature limits.	Maximum temperature recorded during heat cycles.	Connector temperatures remain within specified limits relative to reference conductor temperature.
**Statistical Resistance Factor Analysis**	Evaluate scatter and changes in connector resistance factor across cycles.	Statistical analysis of resistance factors (e.g., δ, β, λ) calculated for 1000 heat cycles.	Resistance scatter and changes remain within specified thresholds; no significant drift or variability over test duration.

**Table 3:** Summary of the main tests specified in IEC 60502-4.

Test Name	Requirements	Testing Techniques/Procedure	Results/Criteria
**AC Voltage Withstand Test**	Test voltage of 4.5 $U_{0}$ applied for 5 min for AC or 4 $U_{0}$ for 15 min for DC.	Apply specified voltage across the joint in air or under test conditions (e.g., wet conditions for terminations).	No visible breakdown or disruptive discharges; insulation integrity must be maintained.
**PD Test**	Maximum partial discharge level: 10 pC at 1.73 $U_{0}$.	Measure partial discharges using sensitive instruments while applying voltage.	Partial discharge should not exceed 10 pC to ensure insulation effectiveness.
**Lightning Impulse Test**	10 positive and 10 negative impulses applied at rated BIL.	Use impulse generator to apply lightning impulse voltages across the joint. Test conducted at ambient and elevated temperatures.	No visible damage or insulation failure during or after the test.
**Thermal Cycle Test**	60 thermal cycles at rated current and voltage.	Heat the conductor to simulate operational thermal stress; cooling occurs between cycles.	No significant changes in joint resistance or mechanical integrity after cycles.
**Short-Circuit Test**	Current applied to raise conductor temperature to maximum permissible level.	Simulate fault currents by applying short-circuit conditions to the joint. Measure temperature rise and visual appearance post-test.	No visible damage to the joint or accessory, and mechanical integrity must be maintained.
**Humidity or Environmental Test**	Joint exposed to humid conditions or submersion while testing voltage withstand capabilities.	Submerge joint or expose it to a controlled humid environment while performing AC or DC voltage withstand tests.	No water ingress, insulation breakdown, or partial discharge anomalies detected.
**Mechanical Tensile Test**	Force applied based on conductor size and type, as specified in the standard.	Apply mechanical force on the joint or termination to simulate mechanical stress under operational conditions.	No slippage, detachment, or mechanical failure observed during or after the test.
**Surface and Corona Test**	Monitor corona discharge and surface tracking under operational voltage.	High-sensitivity instruments used to detect corona discharges and surface tracking across the insulation material.	No significant corona discharge or surface degradation observed.

**Table 4:** Summary of tests from IEC 61442.

Test Name	Requirements	Technique/Procedure	Expected Results
AC Voltage Test	Test accessories in dry and wet conditions, maintain clean surfaces, comply with IEC 60060-1.	Voltage is applied as specified; wet tests involve immersing samples in water or exposing them to controlled rainfall.	No breakdown or flashover.
Impulse Voltage Test	Install samples with specified orientation and comply with IEC 60230. Temperature requirements for conductor must be stabilized to test above-normal operating temperatures.	Apply impulse voltage between one conductor and the others grounded. Conduct tests in line with IEC 60230.	No insulation puncture or damage.
PD Test	Required for extruded insulation cables with semi-conducting screens. Comply with IEC 60270 and IEC 60885-3. Conduct test at elevated temperature (5 K to 10 K above operating).	Measure partial discharges at specified test voltages. Heat conductors for stabilization before testing.	Discharges below specified threshold (e.g., 10 pC for certain tests).
Thermal Short-Circuit Test	Accessories must be connected to short-circuit generators. Test voltage, current, and duration are agreed between customer and manufacturer.	Apply short-circuit currents for specified durations. Measure temperature before and after tests.	No mechanical or electrical failure of cable joint or accessory.
Dynamic Short-Circuit Test	For joints designed for high peak currents (e.g., >80 kA for single-core cables). Use suitable test loops and follow manufacturer’s instructions.	Install joints in cable loops, energize with short-circuit generator. Test dynamic thermal stability and mechanical anchorage.	No displacement, deformation, or functional failure.
Heating Cycles Voltage Test	Prepare samples as per manufacturer’s recommendations; immerse or expose them to heating and cooling cycles.	Cycles include steady heating above the maximum operating temperature followed by cooling. For water immersion tests, damage to oversheath may be introduced intentionally to test water ingress resistance.	Stable performance without insulation damage, flashover, or water ingress.
Humidity and Salt Fog Test	Apparatus must simulate humid/saline conditions per manufacturer’s recommendations.	Expose samples to salt fog or humidity, energize accessories at rated voltages, and monitor for flashover or performance degradation.	No breakdown, flashover, or significant degradation under simulated conditions.
Mechanical Impact Test	Samples mounted per the standard must withstand repeated mechanical impacts.	Subject samples to repeated impacts under specified conditions, maintaining operating voltage during tests.	No damage affecting functionality or safety.

**Table 5:** Main tests from IEEE Std. 592.

Test Name	Purpose	Technique/Procedure	Acceptance Criteria
**Shield Resistance Test**	Ensure insulation shield provides low resistance, limiting hazardous voltages.	Measure shield resistance using the voltmeter–ammeter method with AC or DC current applied at 1.0 ± 0.2 mA.	Resistance ≤5000 Ω for standard measurements; ≤10,000 Ω if joint geometry prevents direct measurement at the center.
**Simulated Touch Current Test**	Demonstrate the shield design limits hazardous current during accidental contact.	Apply AC voltage across the joint. Measure touch current through a 1000 Ω resistor using a circumferential electrode placed at specific points.	Simulated touch current ≤1 mA under maximum voltage rating of the accessory.
**Fault-Current Initiation Test**	Verify the shield’s ability to sustain and reinitiate fault currents under fault conditions.	Insert a fault rod into the accessory and apply fault current (10,000 A rms). Test is repeated to simulate a second fault.	Fault current must initiate within 3 s and sustain ≥7500 A rms for 10 cycles in the first initiation. Subsequent attempts must not fail due to excessive shield erosion or damage.

**Table 6:** Tests specified in BS HD 629.1.

Test Name	Purpose	Technique/Procedure	Acceptance Criteria
AC Voltage Withstand Test	Validate insulation strength under high voltage stress.	Apply AC voltage (4.5 $U_{0}$) for 5 min.	No breakdown or flashover observed.
PD Test	Ensure minimal insulation degradation during operation.	Measure partial discharge at 2 $U_{0}$ using calibrated detection instruments.	Maximum discharge ≤10 pC.
Impulse Voltage Test	Simulate lightning or switching surges to test insulation durability.	Apply 10 positive and 10 negative impulses at rated BIL.	No breakdown or flashover observed during or after the impulses.
Thermal Short-Circuit Test	Confirm cable joint performance under short-circuit conditions.	Apply short-circuit current to raise the conductor to the specified temperature.	No significant mechanical or electrical damage to the joint.
Dynamic Short-Circuit Test	Assess mechanical stability and thermal performance under fault conditions.	Apply a single short-circuit at the specified peak current (e.g., 80 kA or higher).	No breakdown or damage to joint structure.
Heating Cycle Voltage Test	Evaluate joint endurance under repeated thermal and electrical stresses.	Conduct 126 heating cycles at 2.5 $U_{0}$ in air or water, depending on the accessory type.	No breakdown or significant deterioration in performance.
Visual Examination	Inspect for physical integrity, moisture ingress, and material degradation after testing.	Examine joints and document findings per Annex C of the standard.	All components must retain structural and functional integrity.
Water Immersion Test	Verify joint’s water resistance in submerged conditions.	Submerge the joint in water and perform voltage withstand and heating cycle tests.	No water ingress, breakdown, or insulation resistance issues detected.
Impact Test	Confirm mechanical resistance to external impacts.	Apply mechanical impact at ambient temperature and measure insulation resistance.	Insulation resistance ≥1000 MΩ conductor-to-screen, ≥50 MΩ screen-to-water.

## Data Availability

No new data were created or analyzed in this study.

## Abbreviations

The following abbreviations are used in this manuscript:

AI	Artificial Intelligence
ANN	Artificial Neural Network
CJ	Cable Joint
DF	Dissipation Factor
FEA	Finite Element Analysis
HV	High Voltage
ML	Machine Learning
MV	Medium Voltage
PDC	Polarization and Depolarization Current
PD	Partial Discharge
TDR	Time Domain Reflectometry
VLF	Very Low Frequency
